# HER Tyrosine Kinase Family and Rhabdomyosarcoma: Role in Onset and Targeted Therapy

**DOI:** 10.3390/cells10071808

**Published:** 2021-07-16

**Authors:** Carla De Giovanni, Lorena Landuzzi, Arianna Palladini, Giordano Nicoletti, Patrizia Nanni, Pier-Luigi Lollini

**Affiliations:** 1 Laboratory of Immunology and Biology of Metastasis, Department of Experimental, Diagnostic and Specialty Medicine (DIMES), Alma Mater Studiorum University of Bologna, 40126 Bologna, Italy; arianna.palladini@unibo.it (A.P.); patrizia.nanni@unibo.it (P.N.); 2 Laboratory of Experimental Oncology, IRCCS Istituto Ortopedico Rizzoli, 40136 Bologna, Italy; lorena.landuzzi@ior.it (L.L.); giordano.nicoletti@fastwebnet.it (G.N.)

**Keywords:** rhabdomyosarcoma, HER2, EGFR, targeted therapy, CAR-T, precision medicine

## Abstract

Rhabdomyosarcomas (RMS) are tumors of the skeletal muscle lineage. Two main features allow for distinction between subtypes: morphology and presence/absence of a translocation between the PAX3 (or PAX7) and FOXO1 genes. The two main subtypes are fusion-positive alveolar RMS (ARMS) and fusion-negative embryonal RMS (ERMS). This review will focus on the role of receptor tyrosine kinases of the human epidermal growth factor receptor (EGFR) family that is comprised EGFR itself, HER2, HER3 and HER4 in RMS onset and the potential therapeutic targeting of receptor tyrosine kinases. EGFR is highly expressed by ERMS tumors and cell lines, in some cases contributing to tumor growth. If not mutated, HER2 is not directly involved in control of RMS cell growth but can be expressed at significant levels. A minority of ERMS carries a HER2 mutation with driving activity on tumor growth. HER3 is frequently overexpressed by RMS and can play a role in the residual myogenic differentiation ability and in resistance to signaling-directed therapy. HER family members could be exploited for therapeutic approaches in two ways: blocking the HER member (playing a driving role for tumor growth with antibodies or inhibitors) and targeting expressed HER members to vehiculate toxins or immune effectors.

## 1. Introduction

Rhabdomyosarcomas (RMS) are tumors of the skeletal muscle lineage, mainly occurring in children and adolescents. RMS are identified by the expression of markers of myogenic differentiation such as desmin, myogenin and MyoD1. The classification of RMS was initially based on pathological features (alveolar versus embryonal-like pattern morphology). Heterogeneity in histology, biomarkers, molecular driver events and clinical outcome led to a reclassification of RMS subtypes [1,2,3,4] that takes into account the molecular events. In fact, the study of gene expression profiles highlighted a major role for the presence/absence of a translocation between PAX3/7 and FOXO1 genes, leading to a major dichotomy between fusion-positive RMS and fusion-negative RMS [1,5]. A large genomic characterization of 641 RMS cases was designed to further refine risk stratification based on additional molecular alterations [6].

According to the 2020 WHO classification, pediatric RMS can be subdivided into three subtypes: alveolar (ARMS), embryonal (ERMS) and spindle cell/sclerosing (SSRMS) [3]. A fourth subtype, pleomorphic RMS (PRMS), only occurs in adults. ARMS and ERMS are the major subtypes, both with distinctive features. ARMS are defined as PAX3/7–FOXO1 fusion-positive, show a strong myogenin expression (>50% tumor nuclei) and have an unfavorable outcome. ERMS are fusion-negative, have complex genetic changes and mainly have a favorable outcome. The prognosis of ERMS, however, worsens if the tumor arises in unfavorable sites (such as extremities and others) or in patients with high-stage metastatic disease [2,4]. Additional histochemical markers can help RMS diagnostics, such as positivity for AP2β and P-cadherin in ARMS and for the epidermal growth factor receptor (EGFR) and fibrillin-2 in ERMS [3,7]. SSRMS is a rare heterogeneous group with three alternative molecular events: MYOD1 mutations, translocations involving VGLL2/NCOA2 or rearrangements of TFCP2. MYOD1-mutated and TFCP2-rearranged SSRMS have a bad prognosis.

The main oncogenic driver of fusion-positive RMS is the product of fusion itself (the PAX3/7–FOXO1 chimeric transcription factor), but a cooperating event is needed, such as gene amplifications (MYCN, CDK4 or MIR-17–92) or deletions (CDKN2A, loss of heterozygosity in 11p15.5) [8]. Mutations in BCOR (6% of cases), NF1 (4%), TP53 (4%) and PIK3CA (2%) were also found in fusion-positive RMS [6]. PAX3/7–FOXO1 targets include receptor tyrosine kinases (RTK), which are consequently overexpressed and actively signaling along the RAS/Phosphatidylinositol 3-kinase (PI3K) axis [9].

Fusion-negative RMS have a higher degree of aneuploidy and a heterogeneous mutation burden comprising of coexisting and alternative mutations in RTK/RAS/PI3K pathway. Activation of the RAS pathway can be found frequently and can be caused by mutations of RAS itself (14–54% of cases) or by alternative events, either upstream RAS (such as mutations in RTK) or downstream RAS (such as BRAF) [8,10]. More than half of fusion-negative RMS showed mutation of any RAS pathway member [6]. Additional relevant mutations in BCOR (15%), NF1 (15%) and TP53 (13%) were found in fusion-negative RMS [6]. A major hallmark of fusion-negative RMS is the loss of heterozygosity at 11p15 or uniparental paternal disomy of the entire chromosome 11 [10]. The 11p15 region contains various genes, including HRAS and insulin-like growth factor 2 (IGF2). IGF2 is consequently overexpressed due to the loss of imprinting. Loss of heterozygosity at 11p15 and activation of RAS pathway are early events in the genesis of fusion-negative RMS [11].

RMS risk stratification is based on postsurgical staging and clinical group classification (as defined by the Intergroup Rhabdomyosarcoma Study) [1]; it takes into account age, site of tumor, presence/absence of node involvement or metastases and incorporates the presence/absence of a fusion event. According to the European Pediatric Soft Tissue Sarcoma Group, high risk RMS group comprises stage II–III ERMS at unfavorable site, stage I–III ERMS at <10 years of age and ARMS without node involvement. The very high risk group consists of ARMS with node involvement or metastases [4]. TP53 mutations were associated with worse outcome in both fusion-positive and fusion-negative RMS [6] and should be included in future risk evaluation.

For high and very high-risk RMS new therapeutic strategies are urgently needed [12,13,14]. New therapeutic targets can be identified through the study of oncogenic drivers causing RMS. This review will focus on RTK of the HER family, addressing their role in the onset of RMS and therapeutic targeting strategies.

## 2. HER Family

**Figure 1:**
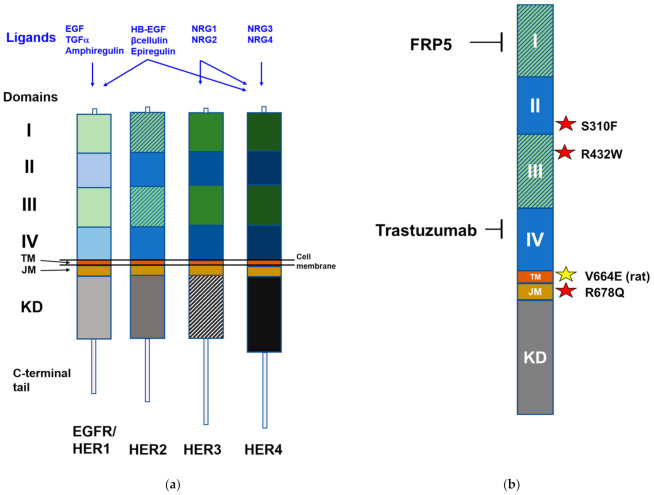
(**a**) HER family members. Main ligands are reported in blue. Abbreviations: EGF, epidermal growth factor; HB-EGF, heparin-binding epidermal growth factor; JM, juxtamembrane; KD, kinase domain; NRG, neuregulin; TGFα, transforming growth factor-α; TM, transmembrane. Domains I and III (leucin-rich) are ligand-binding domains; domain II (cysteine-rich, as domain IV) participates in dimer formation. Kinase domain is involved in signal transduction. HER2 has no ligand (domains I and III shaded). HER3 has an impaired kinase domain (shaded) [16]. (**b**) HER2. Binding sites of monoclonal antibodies trastuzumab and FRP5 are reported along with mutations observed sporadically in human rhabdomyosarcomas (red stars). HER2/neu mutation driving the murine RMS model (yellow star) is V664E of the rat sequence, corresponding to mutation V659E of the human sequence [18].

The HER family belongs to the superfamily of membrane RTK [15] and is composed of four members; EGFR (also named HER1), HER2 (also named HER2/neu, with reference to the homologous neu rat gene), HER3 and HER4 (Figure 1a) [16]. Alternative names are ERBB1, ERBB2, ERBB3 and ERBB4, respectively. Each member of the HER family is composed of four extracellular domains, a transmembrane domain and two cytoplasmic domains (juxtamembrane and kinase domains). HER RTK function as homo- or heterodimers. HER2 has no ligand but is the preferred partner of other HER members. HER3 has little kinase activity. HER2–HER3 is the most active heterodimer. HER family members can bind a complex system of ligands, such as EGF and TGF-α for EGFR or neuregulins for HER3 and HER4 (Figure 1a) [16]. Binding to the specific growth factor promotes dimerization, through a conformational change that exposes the dimerization arms in extracellular domain II. Then, cross-phosphorylation of the cytoplasmic tyrosine kinase domains initiates the downstream signaling cascade. Signaling networks of HER members includes several interconnected and overlapping pathways (such as the PI3K/AKT/mTOR, the RAS/RAF/MEK/ERK1/2 and the phospholipase C pathways), which drive the signaling for cell proliferation and survival [16,17].

HER family members are classified as oncogenes, since they can become constitutively active (and therefore constitutively signaling for proliferation and survival) upon point mutation, truncation or gene amplification [16]. HER2 overexpression (caused by gene amplification) leads to HER2–HER2 constitutively signaling homodimers. Actually, increased expression of any RTK member can generally lead to the formation of homodimers even in the absence of ligands, triggering signaling cascade [15].

In various solid cancers, gene alterations in HER members play a driver role causing tumorigenesis, tumor growth and progression, and also affecting antitumor immune responses [17]. Somatic alterations of EGFR are found in many lung cancer cases [16]. HER2 amplification is found in about 20% of breast cancers and in several other solid tumors with variable frequencies [19]. Somatic mutations of HER2 are found by sequencing large series of tumors, see for example the catalogue of somatic mutations in cancer (COSMIC project) [20]. HER2 point mutations are found across all exons (with some hotspots) and are mostly not associated with concurrent gene amplification. Most HER2 mutations are likely driver alterations and are found at low frequencies (1–3%) across multiple cancer types [19,21].

At the beginning of the millennium, the finding that HER2-overexpressing breast cancer could be targeted with the anti-HER2 monoclonal antibody trastuzumab, combined with chemotherapy, resulting in a therapeutic improvement [22] paved the way to HER2-targeted therapies. Trastuzumab acts through a multiplicity of mechanism of actions, including direct antiproliferative ability and antibody-dependent cellular cytotoxicity [23]. Approximately at the same time the anti-EGFR kinase inhibitor gefitinib showed antitumor activity against EGFR-mutated lung cancer [16]. In the following two decades, a plethora of antibodies and kinase inhibitors against EGFR and HER2 showed therapeutic activity and were incorporated into clinical treatment for lung, breast, gastric and colorectal cancers, as well as other solid tumors [16].

Some issues concerning HER2-targeted therapy remain open. Data suggest that HER2 mutations can confer sensitivity to HER2 targeted drugs; therefore, HER2-targeted approaches could be extended as personalized therapies to all patients whose non-breast solid tumor carries a sporadic HER2 mutation [21]. However, the therapeutic response of patients with amplified versus mutated HER2 tumors remains to be studied in depth [19].

A recent development is the immune targeting of HER family members as tumor-associated antigens by chimeric antigen receptors (CAR) transduced into immune effectors [24].

## 3. HER Family in Myogenesis

EGFR is a known marker of human adult muscle stem cells (satellite cells); with other markers (such as CD56/NCAM), it allows the isolation of adult stem cells from muscles [25,26]. EGFR is a key regulator of myoblast differentiation: its downregulation triggers the differentiation program [27]. EGF stimulates asymmetric cell division, so EGFR is a determinant of cell fate, not acting as a mitogen for satellite cells [28].

HER2, HER3 and HER4 and multiple neuregulin isoforms are natively expressed by skeletal muscles at the neuromuscular junction and by myoblasts in culture [29,30]. HER2 (and other HER family members) appear on satellite cells early during activation and, through the MEK pathway, mediate an antiapoptotic survival signal [30]. HER3 is upregulated after a program of progressive resistance training [29]. Neuregulins, ligands of the HER2/HER4 dimer, are so called for their role in the development of the nervous system and in adult brain homeostasis, but they likely also play a role in skeletal muscle. HER2 and HER3 are upregulated in skeletal muscle after denervation suggesting that neuregulin NRG1 might have an anti-atrophic role in denervated muscle [31].

HER2 and HER4 are crucial for cardiac development and function. Embryonic lethality with cardiac defects was observed in knockout-mice [32]. They also play some role in the muscle homeostasis. NRG1 regulates the heart stress response [33] and induces cardiomyocyte proliferation [34,35]. A well-known side effect of trastuzumab anti-HER2 therapy combined to anthracycline is actually cardiac systolic disfunction in 27% of patients [33].

## 4. Expression of HER Family Members in Human RMS Subtypes

The expression of EGFR and HER2, as evaluated via immunohistochemistry, in the main subtypes of RMS is summarized in Table 1. EGFR was found in the majority of ERMS, at quite strong expression levels, while ARMS only sporadically expressed it. The higher EGFR expression by ERMS versus ARMS was also observed at the mRNA level [36]. The expression of EGFR was proposed as an additional diagnostic marker for ERMS [3,37]. HER2 was expressed only by a fraction of RMS, with a slight prevalence for ARMS [38]. Both EGFR and HER2 were phosphorylated in RMS, while normal skeletal muscle did not show any phosphorylated form of these RTK [39]. To the best of our knowledge, the expression of HER3 and HER4 has never been studied using immunohistochemistry in the RMS series.

Through the study of publicly available microarray experiments, the HER family expression in RMS was compared to that of normal muscle [40]. EGFR expression by ERMS was higher than that of normal muscle. RMS overexpressed HER3 versus normal muscle, with a higher HER3 expression in ARMS than in ERMS. HER4 was downmodulated in RMS versus normal muscle.

## 5. The Role of HER Family in the Onset and Malignancy of RMS

### 5.1. Human RMS

No amplification of the EGFR gene was reported via FISH analysis over 66 RMS cases (Table 1) [38]. The absence of events concerning EGFR was then confirmed in a large RMS series through genome and transcriptome sequencing [8]. Therefore EGFR is not supposed to play a causal role in oncogenic transformation originating RMS. Its expression in most ERMS is likely reminiscent of the differentiative myogenic process [28].

The search for amplification or mutation in HER2 yielded some interesting results. Amplification was sporadically reported in small series of ERMS [43,44]. The study of a large RMS series (147 cases) found no event in fusion-positive RMS, while an expressed mutation of HER2 was found in two cases of fusion-negative RMS (mutations R678Q or S310F), corresponding to 1.4% of total RMS cases (3.2% of fusion-negative RMS cases) [8]. Among RMS cell lines listed in the somatic mutation COSMIC database, only TE-441-T (classified as ERMS, even though no characterization is available, see [45]) carries a mutation in the HER2 gene (R432W) [20]. Therefore, for a small fraction of fusion-negative RMS cases the somatic mutation in HER2 gene can be an oncogenic driver (Figure 1b).

A role for HER3 in RMS differentiation is suggested by two studies. Both ARMS and ERMS cell lines responded to glial-derived growth factor 2 (a specific ligand of HER3 that stimulates normal myogenesis) with an increased myogenic differentiation, in the absence of effects on cell growth [46]. Gene-expression profiling of clones of the ERMS RD cell line with high- versus low-differentiative ability showed that HER3 is expressed only by the clone that maintains (at least partially) the ability to differentiate in vitro [47].

HER3 could also play a protumoral role. The HER3 zebrafish homologue (HES3) is a target gene of the PAX3–FOXO1 chimeric transcription factor resulting from translocation and HES3/HER3 overexpression in fusion-positive RMS was associated with a significantly reduced survival [48]. In the ERMS RD cell line, the induced overexpression of HER3 caused increased cell growth while HER3 silencing determined a decreased growth [40]. The study of an RMS-related miRNA signature, common to all RMS subtype [49], showed that a commonly downregulated miRNA in RMS is the oncosuppressor miR-22. HER3 transcript contains in its 3′UTR a functional response element to miR-22. The upregulation of HER3 is a mechanism of primary resistance to MEK inhibitors shown by RMS [49].

### 5.2. HER2-Driven Murine RMS Model

The oncogenic role that HER2 plays in the transformation towards RMS is proven by a HER2-driven murine model of RMS. A transgenic rat HER2/neu allele, activated by point mutation V664E and expressed under the control of the MMTV-LTR promoter if coupled to an inactivated p53 tumor suppressor allele, caused high-penetrance genitourinary RMS in male mice [50]. Tumors showed typical markers of RMS (desmin, myosin and a high expression of IGF2) and histologically resembled ERMS. Therefore, in this RMS model (named p53neu), two events were required to originate a fusion-negative RMS: an oncogenic driver such as the activated HER2 and the loss of p53 tumor suppressor gene (the latter determining genomic instability). In fact, the loss of p53 was needed in combination with other events in several other mouse models of both fusion-positive and fusion-negative RMS [45,51].

The HER2-driven RMS murine model has an advantage over other RMS murine models due to the predictable site of onset. This allowed us to evaluate the preventive efficacy of active or passive immune approaches and to study the early molecular events leading to RMS genesis. Active prophylactic vaccination with an interleukin 12-engineered allogeneic HER2-positive cell vaccine delayed RMS onset, due to the production of anti-HER2 antibodies and a sustained IFN-γ response (both local and systemic [52]). The administration of anti-IGFs antibodies, as well as the autochthonous induction of anti-IGF2 antibodies by DNA vaccines, also succeeded in the specific delay of RMS onset [53].

HER2-driven RMS are triggered at the genitourinary site of male mice by the coincidental increased expression of HER2 and the under-expression of p53. The two genetic alterations foster p53 loss, IGF2 autocriny and overexpression of p19Arf and p21Cip1 [54].

From HER2-driven murine RMS several cell lines were derived: they showed a low expression of HER2/neu but were tumorigenic and metastatic as well [50,55]. RMS p53neu cell lines also shared with human RMS cell lines the overexpression of connective tissue growth factor, which exerts an antiapoptotic role [56].

## 6. HER Family Members as Therapeutic Targets

### 6.1. Antibodies and Inhibitors in Preclinical Models

For several human cell lines of the two main RMS subtypes, data on the expression of HER family members are available in the literature (Table 2). Some cell lines previously considered derived from RMS have been reclassified as deriving from other tumor types (such as A204) or from contamination with other RMS cell lines (such as TE-671) [45,57], and therefore have been omitted in this review.

RMS cell lines were used as models for functional studies of the role played by HER family members in tumor proliferation and their possible use as therapeutic targets for RMS. In normal as well as neoplastic myogenic models, proliferation can antagonize differentiation [58], and probably vice versa. Therefore, studies aimed at interfering with the activity of RTK (such as HER members) often investigated the effects on both proliferation and differentiation.

EGFR is expressed at high levels by many ERMS cell lines, the most studied of which is RD (Table 2). Among ARMS, the only known expresser is the PAX3–FOXO1 fusion-positive RH30 cell line. HER3 is expressed by most RMS cell lines of both main subtypes (Table 2).

The co-expression of different HER family members is important since the heterodimers formed between EGFR or HER2 with HER3 could signal. Phosphorylated isoforms were observed for HER family members when expressed in RMS [49,63,64], suggesting their active signaling activity in RMS.

Strategies to suppress EGFR activity through the use of neutralizing antibodies [36,64,65] and transduction of the antisense construct [71] generally caused an impairment of the proliferative ability of EGFR-positive RMS cells, with low/null effect on myogenic differentiation.

Anti-EGFR monoclonal antibody cetuximab and the EGFR inhibitor erlotinib decreased growth of RD cells in vitro (through Akt downregulation), and in vivo as xenotransplants, but they did not affect other EGFR-positive RMS cell lines [64]. Therefore, only a subset of RMS could rely upon EGFR for growth-promoting signaling. AKT1 inhibitors showed therapeutic potential, even in RAS-mutated RMS such as RD.

In conclusion, data from preclinical studies show that EGFR is mainly expressed by ERMS cell lines, as well as by primary tumors. In some ERMS (but not all), EGFR can contribute to tumor growth.

The HER2 expression level detected in RMS cells by cytofluorometric analysis was sizeable, but it was at least two orders of magnitude lower than that reported for HER2-overexpressing SK-OV-3 human carcinoma cells [66]. In breast cancers, it is known that anti-HER2 approaches (such as antibodies or inhibitors) can be effective against HER2-overexpressing (and generally HER2-amplified) tumors, but not against cells with normal levels of HER2 [23]. No study was performed to assess RMS sensitivity to the anti-HER2 monoclonal antibody trastuzumab.

The few cases of breast cancer with HER2 mutations (about 1.6%) are studied as candidates for anti-HER2 inhibitors [16]. This approach could be extended to other tumors presenting HER2 driving mutations, including the few cases of fusion-negative RMS [8]. The only RMS cell line with a HER2 mutation known is TE-441-T. The study of the sensitivity of this cell line to the wide panel of anti-HER2 inhibitors available could shed some light on whether sporadic RMS cases with HER2 mutation events could be eligible for anti-HER2 approaches.

HER3 is expressed by most RMS cell lines [67]. Some ARMS cell lines, showing an abundant expression level of HER3 and its phosphorylation, were chosen to test the efficacy of patritumab, an anti-HER3 monoclonal antibody, alone or in combination with erlotinib, an anti-EGFR inhibitor, or with standard cytotoxic agents. Patritumab did not significantly modify ARMS tumor growth when given alone or in combined therapy [67].

HER family members can crosstalk with other RTK members, being involved in the onset of resistance to treatments directed against other receptors for growth factors. The IGF1 receptor (IGF1R) is supposed to play a growth-sustaining role in RMS [58] and was targeted by specific inhibitors. The acquisition of resistance to anti-IGF1R BMS-536924 was correlated with overexpression of EGFR by RMS cells [72]. In a murine model of ARMS, the resistance to anti-IGF1R NVP-AEW541 correlated with HER2 overexpression [73]. In the murine HER2-driven RMS model, the selection of IGF1R-overexpressing clones was accompanied by a decreased HER2 expression [53]. The crosstalk between IGF1R and HER2 can rely upon heterodimer formation between the two RTK [15]. Crosstalk amongst RTK suggests that inhibitor-based therapies should simultaneously block different RTK with combined inhibitors.

### 6.2. Inhibitors in Clinical Trials

A phase 1 clinical study with the EGFR inhibitor erlotinib combined to temozolomide on refractory pediatric solid tumors (including RMS) allowed us to identify the recommended dose of erlotinib [74]. The few cases of RMS included in the study showed low and heterogeneous EGFR expression at immunohistochemistry. A phase 2 trial was launched (NCT02689336), but was later withdrawn. Moreover, a phase 2 trial is currently running with the pan-HER inhibitor afatinib [13].

In targeting therapy, inducing a significant antitumor effect requires the choice and neutralization of a driving target, and not simply of a highly expressed tumor-associated antigen. Preclinical data indicate that HER family members are differently expressed in the RMS of the two main subtypes and that only matching their expression and, even more importantly, their driving activity with the proper neutralization approach could lead to a significant therapeutic result. Due to the paucity of RMS cases, such precision medicine approach will make trials more and more difficult. Complexity increases when considering that different HER2 mutations could have differential sensitivity to inhibitors [75]. An agnostic approach across multiple cancer types bearing different HER2 mutations has been proposed. The most potent HER2 mutant-selective inhibitor found was poziotinib [75]: the effects of such an inhibitor on HER2-mutant ERMS should be studied. A phase 3 clinical trial in which the molecular profiling of advanced sarcoma will guide the choice of the appropriate targeted therapy is recruiting (NCT03784014): a patient with sarcoma presenting EGFR or HER2 molecular drivers will be treated with the dual EGFR/HER2 tyrosine kinase inhibitor lapatinib.

### 6.3. Immunotoxins

The expression of HER family members in RMS cells was exploited to vehiculate toxic agents. This approach could in principle be active even when target antigens are not essential for tumor growth.

Antibodies or single chain variable fragment (scFv) directed against EGFR were conjugated with a toxin fragment like saporin S6 [66] or with a granzyme B mutant [61]. Alternatively, the ligand EGF and a second targeting to membrane urokinase-type plasminogen activator receptor were conjugated with the Pseudomonas exotoxin A [69]. All of these studies showed that EGFR-directed conjugates were able to specifically target EGFR-positive RMS cells, causing decreased cell growth and increased apoptosis.

Immunotoxins targeting HER2 or HER3 did not cause any effect on growth or differentiation of RMS cells [66]. The authors attributed the different efficacy of EGFR versus HER-2/HER-3 immunotoxins to the different endocytic routing of HER receptors, rather than to expression levels of the various target antigens [66].

### 6.4. Chimeric Antigen Receptors

HER family members expression via RMS could be exploited for use in the targeting of immune cells to tumors by chimeric antigen receptors (CAR). CAR constructs code for a single molecule composed of an extracellular scFv domain recognizing a tumor-associated surface antigen and a CD28.ζ endodomain. Other immunostimulating domains can also be included [76,77]. CAR constructs transduced into autologous T cells generate CAR-T cells targeting tumor cells positive for the tumor-associated antigen. Impressive therapeutic responses were obtained with CAR-T targeting antigens expressed by leukemias and lymphomas, such as CD19 [76,77]. Less satisfying results were obtained against solid tumors [24]. Combinations of CAR-T therapy with checkpoint inhibitors have been proposed to overcome the suppressive microenvironment of solid tumors [78]. The main drawbacks of CAR-T strategies are the costs and logistics of a personalized therapy on the one hand and the risk of potentially fatal side effects (due to the induction of cytokine storm or to off-tumor, on-target effects [24]).

To obtain HER2-directed CAR (HER2-CAR) T cells, scFv derived from different anti-HER2 monoclonal antibodies were tested against several kinds of HER2-positive carcinomas. Trastuzumab-derived HER2-CAR-T caused severe off-tumor on-target side effects [79]. A better safety profile was obtained with HER2-CAR-T exploiting the scFv derived from the FRP5 anti-HER2 monoclonal antibody. FRP5 binds the extracellular domain I of HER2 (Figure 1b), but binding does not affect tumor proliferation. Therefore, FRP5 mediates a mere targeting to HER2-expressing cells [80].

Besides HER2-expressing carcinomas, HER2-CAR-T cells could target other HER2-positive solid tumors, including sarcomas [81,82,83]. FRP5-derived HER2-CAR-T cells were tested in phase I/II clinical trials against sarcomas with low-to-moderate expression of HER2. HER2-CAR-T cells showed at least a 6-week in vivo persistence and a quite good safety profile [84,85].

FRP5-derived HER2-CAR-T cells were administered to a child with score 3 HER2-positive fusion-negative ARMS, metastatic to bone marrow and refractory to conventional therapy [86]. Multiple infusions of HER2-CAR-T, associated to lymphodepletion, induced remission. At 6 months off-therapy, a relapse in bone marrow was treated with additional infusions of HER2-CAR-T cells combined with the checkpoint inhibitor PD-1 blocking antibody pembrolizumab. A remission was again induced and persisted up to 20 months of follow-up. This study suggests that HER2-CAR-T cells could be therapeutic agents against those RMS (which express HER2).

Besides the already mentioned studies [85,86], two clinical trials currently active and/or recruiting exploit CAR technology to direct T cells against solid tumors (including rhabdomyosarcoma [9]). NCT00902044 is a Phase I trial using HER2-CAR-T cells combined with lymphodepletion against advanced sarcomas. NCT03618381 is a Phase I trial using EGFR-CAR-T cells against relapsed or refractory noncentral nervous system solid tumors.

Alternative effectors to be transduced with CAR constructs could be cytokine-induced killer (CIK) cells that are in vitro-expanded immune effectors exhibiting T and NK phenotypes (so-called T-NK) and a non-MHC-restricted cytotoxic ability [68]. T-NK exhibit minimal alloreactivity, so they can be derived from peripheral blood of haploidentical healthy first-degree relatives through in vitro culture with defined cytokines. HER2-CAR-CIK cells were tested against RMS in preclinical models. HER2-CAR-CIK cells, when compared to non-transduced CIK cells, showed an enhanced antitumor in vitro cytotoxicity against HER2-positive RMS cells [68] and an increased ability to inhibit initial in vivo growth of RH30 xenografts in severely immunodepressed mice [87]; however, they were much less effective when tested against established RH30 xenografts [87].

The engineering of CAR constructs into natural killer (NK) cells could offer some advantages over CAR-T approaches: CAR-NK could constitute “off-the-shelf” products, since they do not require a tight HLA matching nor present the risk of graft-versus-host disease [88]. Since the isolation and transduction of NK cells is difficult and generally unsuccessful, two types of effectors with NK activity were proposed as recipients of CAR construct: NK cells derived from induced pluripotent stem cells or the NK cell line NK92 [88]. NK92 cells were transduced with a FPR5-derived HER2-CAR construct and the HER2-CAR-NK92 obtained were tested in vitro against ARMS cell lines RH30 and RH41 [89]. HER2-CAR-NK92 showed a higher cytotoxicity than NK92 cells.

CAR-modified effectors targeting HER family members for RMS therapy are in their infancy. The optimization of CAR constructs and recipient effector cells, and combination with checkpoint inhibitors to contrast immunosuppressive tumor microenvironment might lead to better antitumor activity.

## 7. Conclusions

In conclusion, different approaches could be evaluated for RMS therapy based on the expression of HER family.

The minority of ERMS that carries a mutant HER2 with driving potential on tumor growth could benefit from HER2 inhibitors, just as proposed for multiple cancer types with HER2 driving mutations. To do this, all RMS should be subjected to genome sequencing (or at least to HER2 sequencing) to identify the tumors that match the therapy. Precision medicine is proceeding along the way of a meticulous molecular characterization exactly to match molecular events and proper therapy. A NCI-MATCH trial, designed to identify molecular therapeutic targets in tumors of different histologies, demonstrated the feasibility of a large national network trial for profiling fresh tumor biopsies and consequently assigning proper targeted therapy [90].

For some ERMS, EGFR could participate in a growth-promoting pathway. However, at the moment, no biomarker predicts which ERMS are sensitive to anti-EGFR approach.

EGFR and HER2 expression on RMS can be exploited to deliver toxins or immune effector cells to tumors. The setup of CAR-directed therapy seems particularly promising, but the immunosuppressive tumor microenvironment likely requires that a combined treatment with checkpoint inhibitors be applied. Data are still very limited, albeit promising. In this area, the assessment of the minimal level of expression of the targeted HER family members remains a critical issue. Preclinical models with RMS cell lines allow for a semiquantitative, highly-sensitive evaluation via cytofluorometric analysis, but tumors are mainly studied via immunohistochemistry [68]. The assessment of the threshold level to gain a significant therapeutic activity should allow for the standardization of a relationship between expression level and biologic response, as was done for HER2-positive breast cancer treatment in the last decades.

## Figures and Tables

**Table 1:** The expression of HER family members in RMS subtypes.

HER Family Member	ARMS	ERMS	Other Subtypes	Intensity at Immuno- Histochemistry	Amplification/Mutation	Reference
EGFR	16%	76%		Moderate to strong	No amplification ^a^ at 7p11.2	[38]
	13%	84%	42% ^b^	Strong in ERMS		[7]
	32%	55%	73% ^c^			[41]
	29%	93%				[37]
HER2	41%	26%			No amplification ^a^	[38]
	6%	6%	27% ^c^			[41]
			70% ^d^			[42]

^a^ fluorescent in situ hybridization (FISH), 66 cases of which 32 ARMS and 34 ERMS. ^b^ fusion-negative ARMS. ^c^ PRMS. ^d^ head and neck RMS (29 cases, of which 18 were ERMS, 10 were ARMS and 1 was PRMS).

**Table 2:** Expression of HER members by human RMS cell lines. References are reported in square brackets. Very high expression: +++, high expression: ++, weak expression: +, borderline expression: +/−, negative: −.

RMS Cell Line	RMS Subtype	RAS Mutation/Translocation	Method	EGFR	HER2	HER3	HER4
RD	ERMS	NRAS Q61H [59]	FC	+++ [36,60,61]	+ [62]	++ [62]	− [62]
			WB	++ ^a^ [63,64]	+ ^a^ [63]	+ ^a^ [40]	
RD/18 ^b^	ERMS	NRAS Q61H [59]	FC	+++ [46,65,66]	+ [46,66]	++ [46,66]	− [46]
			WB			+/− ^a^ [49]	
RD/12 ^c^	ERMS		FC	+++ [62]	+ [62]	− [62]	
RMS-YM	ERMS		FC	+ [60]			
KYM-1	ERMS ^d^		FC	− [60]			
CCA	ERMS	KRAS Q61L [59]	FC	++ [46,65]	++ [46]	++ [46]	− [46]
RH36	ERMS	HRAS Q61K [10]	WB	++ ^a^ [64,67]		+ [67]	
RH4/RH41 ^e^	ARMS	PAX3/FOXO1 [45]	FC	− [46]	+/− 46,68]	+ [46]	− [46]
			WB	++ [67]		++ [67]	
RH5	ARMS	PAX3/FOXO1 [45]	WB	− [67]		+++ [67]	
RH10	ARMS	PAX3/FOXO1 [45,57]	WB	+/− [67]		+++ [67]	
RH18 ^f^	ARMS^f^	PAX3/FOXO1 ^f^ [57]	WB	+++ ^a^ [67]		− [67]	
RH28	ARMS	PAX3/FOXO1 [45,57]	WB	+/− [67]		+/− [67]	
RH30	ARMS	PAX3/FOXO1 [45,57]	FC	++ [36,46,60,69]	+ [46,68]	+ [46]	− [46]
			WB	++ ^a^ [63,67]	+ ^a^ [63]	+ [63,67]	
RC2 or RMZ-RC2	ARMS	PAX7/FOXO1 [70]	FC	− [46,65]	+ [46,65]	++ [46]	− [46]
CW9019	ARMS	PAX7/FOXO1 [45,57]	WB	+ ^a^ [63]	+ ^a^ [63]		

^a^ phosphorylated. ^b^ RD-derived clone with high-differentiation ability. ^c^ RD-derived clone with low-differentiation ability. ^d^ previously classified as fusion-negative ARMS. ^e^ RH4 and RH41 were independently derived from the same tumor. ^f^ a fusion-negative variant of RH18 was also reported [45]. Abbreviations: FC, flow cytometry; WB, Western blot; ERMS, embryonal RMS; ARMS, alveolar RMS.

## Data Availability

Not applicable.
